# Dichlorido(3-phenyl­indenyl­idene)bis­(triphenyl­phosphane)ruthenium(II) tetra­hydro­furan disolvate

**DOI:** 10.1107/S1600536811016692

**Published:** 2011-05-07

**Authors:** Jan W. Bats, Jessica Pérez Gomes, Angelino Doppiu, A. Stephen K. Hashmi

**Affiliations:** a Institut für Organische Chemie, Universität Frankfurt, Max-von-Laue-Strasse 7, D-60438 Frankfurt am Main, Germany; b Umicore AG & Co. KG, Strategic Research and Development, Precious Metals Chemistry, Rodenbacher Chaussee 4, D-63457 Hanau, Germany; c Organisch-Chemisches Institut, Universität Heidelberg, Im Neuenheimer Feld 270, D-69120 Heidelberg, Germany

## Abstract

The Ru^II^ atom in the title compound, [RuCl_2_(C_15_H_10_)(C_18_H_15_P)_2_]·2C_4_H_8_O, has a distorted square-pyramidal conformation. The P and Cl atoms are at the base of the pyramid and the Ru—C_indenyl­idene_ bond is in the axial position. The two Cl ligands and the two phosphane ligands are in *trans* positions. The Cl—Ru—Cl and P—Ru—P angles are 157.71 (2) and 166.83 (2)°, respectively. The two independent tetra­hydro­furan (THF) solvent mol­ecules are disordered. One THF mol­ecule was refined using a split-atom model. The second THF mol­ecule was accounted for by using program *PLATON*/*SQUEEZE* [Spek (2009 ▶). *Acta Cryst.* D**65**, 148–155]. The molecular conformation shows three intramolecular C—H⋯Cl contacts and two C—H⋯π interactions while the crystal packing features an intermolecular C—H⋯Cl contact and two very weak intermolecular C—H⋯π contacts.

## Related literature

For the preparation of the title compound, see: Shaffer *et al.* (2007 ▶). For a related structure, see: Forman *et al.* (2006 ▶). For the treatment of the disordered solvate, see: Spek (2009 ▶).
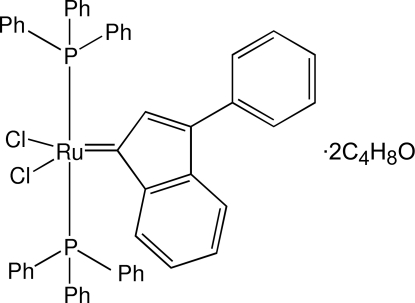


## Experimental

### 

#### Crystal data

[RuCl_2_(C_15_H_10_)(C_18_H_15_P)_2_]·2C_4_H_8_O
*M* *_r_* = 1030.95
Monoclinic, 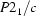

*a* = 17.0955 (6) Å
*b* = 13.6504 (5) Å
*c* = 21.6791 (8) Å
β = 94.229 (1)°
*V* = 5045.3 (3) Å^3^
*Z* = 4
Mo *K*α radiation
μ = 0.52 mm^−1^
*T* = 168 K
0.60 × 0.55 × 0.55 mm

#### Data collection

Siemens SMART 1K CCD diffractometer
Absorption correction: multi-scan (*SADABS*; Sheldrick, 2000 ▶) *T*_min_ = 0.625, *T*_max_ = 0.750
62596 measured reflections
15416 independent reflections
12401 reflections with *I* > 2σ(*I*)
*R*_int_ = 0.046

#### Refinement

*R*[*F* ^2^ > 2σ(*F* ^2^)] = 0.042
*wR*(*F* ^2^) = 0.106
*S* = 0.97
15416 reflections
556 parameters
6 restraints
H-atom parameters constrained
Δρ_max_ = 0.74 e Å^−3^
Δρ_min_ = −0.39 e Å^−3^

### 

Data collection: *SMART* (Siemens, 1995 ▶); cell refinement: *SAINT* (Siemens, 1995 ▶); data reduction: *SAINT*; program(s) used to solve structure: *SHELXS97* (Sheldrick, 2008 ▶); program(s) used to refine structure: *SHELXL97* (Sheldrick, 2008 ▶); molecular graphics: *SHELXTL* (Sheldrick, 2008 ▶); software used to prepare material for publication: *SHELXL97*.

## Supplementary Material

Crystal structure: contains datablocks global, I. DOI: 10.1107/S1600536811016692/si2353sup1.cif

Structure factors: contains datablocks I. DOI: 10.1107/S1600536811016692/si2353Isup2.hkl

Additional supplementary materials:  crystallographic information; 3D view; checkCIF report

## Figures and Tables

**Table 1:** Selected bond lengths (Å)

Ru1—C1	1.8571 (19)
Ru1—Cl2	2.3498 (5)
Ru1—Cl1	2.3639 (5)
Ru1—P1	2.3863 (5)
Ru1—P2	2.4087 (5)

**Table 2:** Hydrogen-bond geometry (Å, °) *Cg*1 and *Cg*2 are the centroids of the C34–C39 and C16–C21 rings, respectively.

*D*—H⋯*A*	*D*—H	H⋯*A*	*D*⋯*A*	*D*—H⋯*A*
C2—H2*A*⋯C28	0.95	2.59	3.271 (3)	128
C2—H2*A*⋯C33	0.95	2.63	3.522 (3)	156
C51—H51*A*⋯C1	0.95	2.59	3.448 (3)	150
C8—H8*A*⋯Cl2	0.95	2.80	3.497 (2)	131
C17—H17*A*⋯Cl2	0.95	2.80	3.640 (3)	148
C33—H33*A*⋯Cl1	0.95	2.66	3.359 (2)	131
C48—H48*A*⋯Cl2^i^	0.95	2.83	3.699 (2)	153
C25—H25*A*⋯*Cg*1^ii^	0.95	2.88	3.727 (3)	149
C30—H30*A*⋯*Cg*2^iii^	0.95	2.98	3.751 (3)	139

Symmetry codes: (i) 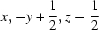; (ii) 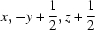; (iii) 
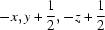
.

**Fig. 1.**
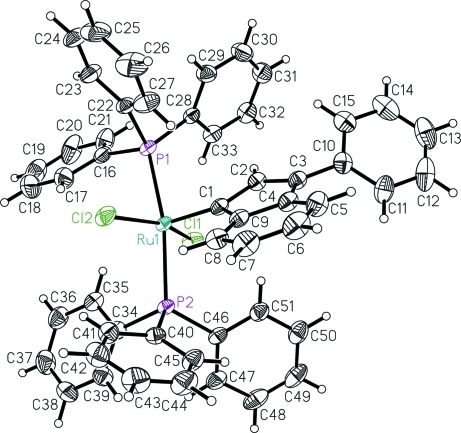
The structure of the title molecule shown with 50% probability displacement ellipsoids. The H atoms are drawn as small spheres of arbitrary radius. The disordered THF solvate molecule has been omitted.

## supplementary crystallographic information

### Comment

The title compound is a precursor to a class of olefin metathesis catalysts (Shaffer *et al.*, 2007).

The Ru^II^ atom has a fivefold coordination of two Cl, two P and one C atoms (Table 1). The coordination corresponds to a distorted square pyramide, with the Ru—C bond in axial position and the P and Cl atoms at the base of the pyramide. The Ru^II^ atom is positioned 0.363 (1)Å above the base plane towards the center of the pyramide and the Cl—Ru—Cl and P—Ru—P angles are 157.71 (2)° and 166.83 (2)° respectively. A very similar coordination has been observed in the crystal structure of a related compound by Forman *et al.* (2006). The sixth coordination site of the Ru^II^ atom is shielded by a phenyl group (distance H35A···Ru1: 2.78 Å). The angle between the planes of the indene group and the phenyl group attached to the indene group is 50.6 (1)°. The molecular conformation shows three intramolecular C—H···Cl contacts with H···Cl distances between 2.66 and 2.80Å and two intramolecular C—H···C_π_ interactions (Table 2). These C—H···C_π_ contacts do not point to the center of the acceptor ring, but the C2—H2A bond mainly points towards the C28—C33 bond and the C51—H51A bond towards atom C1. The indene group is slightly bend (atoms C1, C2 and C3 deviate by 0.105 (3), 0.224 (3) and 0.085 (3)Å respectively in the same direction from the plane of the six-membered ring). This deviation from planarity may result from the involvement of the C2—H2A bond in the intramolecular C—H···C_π_ interaction. The crystal packing shows an intermolecular C—H···Cl contact and two very weak intermolecular C_phenyl_—H···π contacts (Table 2; *Cg*1 and *Cg*2 are the centroids of the C34–C39 and C16–C21 phenyl rings respectively).

### Experimental

The title compound was prepared as described by Shaffer *et al.* (2007). Single crystals were obtained by recrystallization of the compound from THF/2-propanol (1:1).

### Refinement

A disordered tetrahydrofuran molecule was refined with split atoms. The occupancy factor refined to 0.505 (8) for atoms O1, C52, C53, C54 and C55 and to 0.495 (8) for atoms O1', C52', C53', C54' and C55'. Partly occupied C atoms were refined with isotropic displacement parameters. Six distance constrains were applied to the disordered molecule. A grossly disordered second tetrahydrofuran solvate molecule was accounted for by using the program *PLATON*/SQUEEZE (Spek, 2009). The H atoms were positioned geometrically and treated as riding: C_planar_—H=0.95 Å, C_THF_—H=0.99Å and *U*_iso_(H)=1.2*U*_eq_(C).

### Crystal data

**Table:**

[RuCl_2_(C_15_H_10_)(C_18_H_15_P)_2_]·2C_4_H_8_O	*F*(000) = 2136
*M**_r_* = 1030.95	*D*_x_ = 1.357 Mg m^−^^3^
Monoclinic, *P*2_1_/*c*	Mo *K*α radiation, λ = 0.71073 Å
Hall symbol: -P 2ybc	Cell parameters from 8192 reflections
*a* = 17.0955 (6) Å	θ = 3–31°
*b* = 13.6504 (5) Å	µ = 0.52 mm^−^^1^
*c* = 21.6791 (8) Å	*T* = 168 K
β = 94.229 (1)°	Block, black
*V* = 5045.3 (3) Å^3^	0.60 × 0.55 × 0.55 mm
*Z* = 4	

### Data collection

**Table:**

Siemens SMART 1K CCD diffractometer	15416 independent reflections
Radiation source: normal-focus sealed tube	12401 reflections with *I* > 2σ(*I*)
graphite	*R*_int_ = 0.046
ω scans	θ_max_ = 31.1°, θ_min_ = 1.8°
Absorption correction: multi-scan (*SADABS*; Sheldrick, 2000)	*h* = −22→24
*T*_min_ = 0.625, *T*_max_ = 0.750	*k* = −19→19
62596 measured reflections	*l* = −26→31

### Refinement

**Table:**

Refinement on *F*^2^	Primary atom site location: structure-invariant direct methods
Least-squares matrix: full	Secondary atom site location: difference Fourier map
*R*[*F*^2^ > 2σ(*F*^2^)] = 0.042	Hydrogen site location: inferred from neighbouring sites
*wR*(*F*^2^) = 0.106	H-atom parameters constrained
*S* = 0.97	*w* = 1/[σ^2^(*F*_o_^2^) + (0.04*P*)^2^ + 3.6*P*] where *P* = (*F*_o_^2^ + 2*F*_c_^2^)/3
15416 reflections	(Δ/σ)_max_ = 0.003
556 parameters	Δρ_max_ = 0.74 e Å^−^^3^
6 restraints	Δρ_min_ = −0.39 e Å^−^^3^

### Special details

**Table:**

Geometry. All e.s.d.'s (except the e.s.d. in the dihedral angle between two l.s. planes) are estimated using the full covariance matrix. The cell e.s.d.'s are taken into account individually in the estimation of e.s.d.'s in distances, angles and torsion angles; correlations between e.s.d.'s in cell parameters are only used when they are defined by crystal symmetry. An approximate (isotropic) treatment of cell e.s.d.'s is used for estimating e.s.d.'s involving l.s. planes.
Refinement. Refinement of *F*^2^ against ALL reflections. The weighted *R*-factor *wR* and goodness of fit *S* are based on *F*^2^, conventional *R*-factors *R* are based on *F*, with *F* set to zero for negative *F*^2^. The threshold expression of *F*^2^ > σ(*F*^2^) is used only for calculating *R*-factors(gt) *etc*. and is not relevant to the choice of reflections for refinement. *R*-factors based on *F*^2^ are statistically about twice as large as those based on *F*, and *R*- factors based on ALL data will be even larger.

### Fractional atomic coordinates and isotropic or equivalent isotropic displacement parameters (Å^2^)

**Table:**

	*x*	*y*	*z*	*U*_iso_*/*U*_eq_	Occ. (<1)
Ru1	0.244613 (8)	0.311904 (11)	0.096522 (6)	0.02062 (5)	
Cl1	0.15145 (3)	0.28743 (4)	0.01183 (2)	0.02982 (10)	
Cl2	0.33497 (3)	0.26978 (4)	0.17921 (2)	0.03440 (12)	
P1	0.14143 (3)	0.31030 (4)	0.16489 (2)	0.02287 (10)	
P2	0.34194 (3)	0.27407 (4)	0.02525 (2)	0.02216 (10)	
C1	0.25090 (11)	0.44770 (14)	0.09539 (8)	0.0241 (4)	
C2	0.18457 (12)	0.51330 (14)	0.07984 (9)	0.0267 (4)	
H2A	0.1313	0.4931	0.0751	0.032*	
C3	0.20952 (12)	0.60720 (14)	0.07307 (9)	0.0272 (4)	
C4	0.29517 (12)	0.61061 (15)	0.09006 (9)	0.0287 (4)	
C5	0.34622 (14)	0.68880 (17)	0.09833 (11)	0.0384 (5)	
H5A	0.3288	0.7539	0.0901	0.046*	
C6	0.42438 (15)	0.66972 (19)	0.11913 (12)	0.0445 (6)	
H6A	0.4604	0.7225	0.1248	0.053*	
C7	0.44964 (14)	0.57502 (19)	0.13146 (11)	0.0405 (5)	
H7A	0.5030	0.5636	0.1448	0.049*	
C8	0.39771 (12)	0.49547 (17)	0.12464 (10)	0.0317 (4)	
H8A	0.4150	0.4307	0.1343	0.038*	
C9	0.32064 (11)	0.51371 (14)	0.10351 (9)	0.0261 (4)	
C10	0.16009 (13)	0.68942 (14)	0.05032 (10)	0.0312 (4)	
C11	0.18359 (16)	0.75102 (18)	0.00382 (12)	0.0444 (6)	
H11A	0.2336	0.7422	−0.0117	0.053*	
C12	0.1345 (2)	0.8254 (2)	−0.02003 (15)	0.0581 (8)	
H12A	0.1507	0.8661	−0.0523	0.070*	
C13	0.06240 (19)	0.8400 (2)	0.00310 (15)	0.0561 (8)	
H13A	0.0293	0.8915	−0.0126	0.067*	
C14	0.03844 (16)	0.7799 (2)	0.04899 (14)	0.0501 (7)	
H14A	−0.0114	0.7898	0.0647	0.060*	
C15	0.08659 (14)	0.70462 (17)	0.07256 (12)	0.0383 (5)	
H15A	0.0693	0.6632	0.1041	0.046*	
C16	0.10685 (13)	0.18391 (15)	0.16548 (9)	0.0301 (4)	
C17	0.16359 (17)	0.11129 (18)	0.17758 (13)	0.0475 (6)	
H17A	0.2167	0.1293	0.1873	0.057*	
C18	0.1428 (2)	0.0130 (2)	0.17542 (16)	0.0624 (8)	
H18A	0.1817	−0.0360	0.1836	0.075*	
C19	0.0653 (2)	−0.0139 (2)	0.16126 (13)	0.0621 (9)	
H19A	0.0512	−0.0812	0.1591	0.075*	
C20	0.0088 (2)	0.0569 (2)	0.15035 (13)	0.0581 (8)	
H20A	−0.0444	0.0385	0.1415	0.070*	
C21	0.02958 (16)	0.15592 (19)	0.15221 (12)	0.0436 (6)	
H21A	−0.0097	0.2045	0.1443	0.052*	
C22	0.17014 (12)	0.33818 (16)	0.24557 (9)	0.0293 (4)	
C23	0.13841 (16)	0.2885 (2)	0.29402 (11)	0.0426 (6)	
H23A	0.1032	0.2356	0.2855	0.051*	
C24	0.15844 (19)	0.3166 (2)	0.35511 (11)	0.0561 (8)	
H24A	0.1375	0.2818	0.3880	0.067*	
C25	0.20789 (18)	0.3938 (3)	0.36791 (11)	0.0582 (8)	
H25A	0.2202	0.4135	0.4095	0.070*	
C26	0.23976 (18)	0.4430 (3)	0.32049 (12)	0.0579 (8)	
H26A	0.2742	0.4966	0.3295	0.069*	
C27	0.22195 (15)	0.4146 (2)	0.25924 (11)	0.0422 (5)	
H27A	0.2454	0.4478	0.2268	0.051*	
C28	0.05427 (11)	0.38738 (14)	0.14953 (9)	0.0247 (4)	
C29	0.03538 (13)	0.45904 (16)	0.19193 (10)	0.0339 (4)	
H29A	0.0644	0.4628	0.2310	0.041*	
C30	−0.02535 (14)	0.52500 (18)	0.17780 (12)	0.0419 (5)	
H30A	−0.0377	0.5732	0.2072	0.050*	
C31	−0.06805 (13)	0.52036 (18)	0.12068 (11)	0.0386 (5)	
H31A	−0.1087	0.5664	0.1105	0.046*	
C32	−0.05087 (13)	0.44811 (17)	0.07879 (11)	0.0354 (5)	
H32A	−0.0805	0.4440	0.0401	0.042*	
C33	0.00933 (12)	0.38172 (16)	0.09297 (10)	0.0297 (4)	
H33A	0.0201	0.3321	0.0641	0.036*	
C34	0.33981 (12)	0.14104 (14)	0.01386 (9)	0.0264 (4)	
C35	0.27991 (13)	0.08598 (16)	0.03708 (10)	0.0331 (4)	
H35A	0.2399	0.1179	0.0577	0.040*	
C36	0.27840 (16)	−0.01540 (17)	0.03030 (12)	0.0430 (6)	
H36A	0.2380	−0.0525	0.0470	0.052*	
C37	0.33565 (17)	−0.06223 (17)	−0.00069 (13)	0.0468 (6)	
H37A	0.3337	−0.1312	−0.0062	0.056*	
C38	0.39555 (15)	−0.00862 (17)	−0.02357 (12)	0.0413 (5)	
H38A	0.4347	−0.0409	−0.0450	0.050*	
C39	0.39894 (13)	0.09224 (16)	−0.01539 (11)	0.0340 (4)	
H39A	0.4415	0.1283	−0.0297	0.041*	
C40	0.44632 (11)	0.29286 (14)	0.04619 (9)	0.0259 (4)	
C41	0.48345 (13)	0.23486 (16)	0.09260 (10)	0.0321 (4)	
H41A	0.4547	0.1853	0.1118	0.039*	
C42	0.56203 (14)	0.24928 (19)	0.11084 (12)	0.0407 (5)	
H42A	0.5870	0.2092	0.1422	0.049*	
C43	0.60423 (14)	0.3218 (2)	0.08351 (13)	0.0448 (6)	
H43A	0.6579	0.3321	0.0965	0.054*	
C44	0.56822 (14)	0.3789 (2)	0.03751 (12)	0.0442 (6)	
H44A	0.5974	0.4281	0.0184	0.053*	
C45	0.48934 (13)	0.36517 (17)	0.01872 (10)	0.0345 (5)	
H45A	0.4649	0.4053	−0.0129	0.041*	
C46	0.32623 (11)	0.32910 (15)	−0.05201 (9)	0.0256 (4)	
C47	0.35151 (14)	0.28431 (18)	−0.10461 (10)	0.0353 (5)	
H47A	0.3772	0.2226	−0.1015	0.042*	
C48	0.33904 (17)	0.3301 (2)	−0.16197 (10)	0.0462 (6)	
H48A	0.3563	0.2992	−0.1978	0.055*	
C49	0.30199 (15)	0.4196 (2)	−0.16712 (10)	0.0445 (6)	
H49A	0.2938	0.4504	−0.2064	0.053*	
C50	0.27673 (14)	0.46445 (19)	−0.11509 (11)	0.0397 (5)	
H50A	0.2514	0.5263	−0.1185	0.048*	
C51	0.28838 (13)	0.41924 (16)	−0.05767 (10)	0.0317 (4)	
H51A	0.2704	0.4501	−0.0221	0.038*	
O1	0.2419 (5)	0.8257 (8)	0.2921 (5)	0.163 (6)	0.505 (8)
C52	0.1623 (5)	0.8076 (6)	0.2994 (4)	0.074 (2)*	0.505 (8)
H52A	0.1310	0.8658	0.2859	0.089*	0.505 (8)
H52B	0.1549	0.7961	0.3437	0.089*	0.505 (8)
C53	0.1332 (7)	0.7181 (11)	0.2615 (6)	0.119 (4)*	0.505 (8)
H53A	0.1100	0.6692	0.2886	0.143*	0.505 (8)
H53B	0.0928	0.7378	0.2288	0.143*	0.505 (8)
C54	0.2016 (9)	0.6765 (13)	0.2339 (8)	0.164 (6)*	0.505 (8)
H54A	0.1935	0.6725	0.1883	0.196*	0.505 (8)
H54B	0.2154	0.6110	0.2509	0.196*	0.505 (8)
C55	0.2640 (7)	0.7540 (9)	0.2546 (6)	0.120 (4)*	0.505 (8)
H55A	0.3093	0.7196	0.2759	0.145*	0.505 (8)
H55B	0.2826	0.7848	0.2170	0.145*	0.505 (8)
O1'	0.2487 (4)	0.7258 (9)	0.3217 (3)	0.152 (5)	0.495 (8)
C52'	0.1704 (6)	0.7421 (11)	0.3238 (5)	0.130 (4)*	0.495 (8)
H52C	0.1612	0.7982	0.3512	0.156*	0.495 (8)
H52D	0.1445	0.6835	0.3400	0.156*	0.495 (8)
C53'	0.1369 (7)	0.7642 (10)	0.2572 (5)	0.101 (3)*	0.495 (8)
H53C	0.0879	0.7272	0.2468	0.121*	0.495 (8)
H53D	0.1264	0.8351	0.2514	0.121*	0.495 (8)
C54'	0.1986 (5)	0.7322 (7)	0.2205 (4)	0.072 (2)*	0.495 (8)
H54C	0.2135	0.7861	0.1932	0.087*	0.495 (8)
H54D	0.1800	0.6764	0.1941	0.087*	0.495 (8)
C55'	0.2681 (7)	0.7015 (9)	0.2614 (4)	0.100 (3)*	0.495 (8)
H55C	0.2774	0.6302	0.2577	0.120*	0.495 (8)
H55D	0.3157	0.7371	0.2506	0.120*	0.495 (8)

### Atomic displacement parameters (Å^2^)

**Table:**

	*U*^11^	*U*^22^	*U*^33^	*U*^12^	*U*^13^	*U*^23^
Ru1	0.02045 (8)	0.02627 (8)	0.01508 (7)	−0.00202 (5)	0.00099 (5)	0.00112 (5)
Cl1	0.0240 (2)	0.0446 (3)	0.0205 (2)	−0.00430 (19)	−0.00092 (17)	−0.00513 (18)
Cl2	0.0292 (2)	0.0525 (3)	0.0211 (2)	0.0047 (2)	−0.00122 (19)	0.0080 (2)
P1	0.0229 (2)	0.0292 (2)	0.0167 (2)	−0.00177 (18)	0.00305 (18)	0.00171 (17)
P2	0.0206 (2)	0.0281 (2)	0.0179 (2)	−0.00151 (18)	0.00179 (17)	0.00056 (17)
C1	0.0271 (9)	0.0286 (9)	0.0166 (8)	−0.0047 (7)	0.0008 (7)	−0.0006 (7)
C2	0.0253 (9)	0.0284 (9)	0.0264 (9)	−0.0051 (7)	0.0009 (7)	−0.0003 (7)
C3	0.0275 (10)	0.0279 (9)	0.0262 (9)	−0.0060 (7)	0.0028 (8)	−0.0001 (7)
C4	0.0289 (10)	0.0314 (10)	0.0257 (9)	−0.0076 (8)	0.0014 (8)	−0.0001 (7)
C5	0.0380 (12)	0.0351 (11)	0.0414 (12)	−0.0118 (9)	−0.0008 (10)	0.0008 (9)
C6	0.0365 (12)	0.0490 (14)	0.0475 (14)	−0.0217 (11)	−0.0014 (11)	−0.0024 (11)
C7	0.0277 (11)	0.0516 (14)	0.0413 (13)	−0.0117 (10)	−0.0035 (9)	−0.0006 (10)
C8	0.0280 (10)	0.0393 (11)	0.0274 (10)	−0.0045 (8)	−0.0008 (8)	−0.0026 (8)
C9	0.0261 (9)	0.0315 (9)	0.0206 (8)	−0.0069 (7)	0.0014 (7)	−0.0009 (7)
C10	0.0333 (11)	0.0262 (9)	0.0330 (10)	−0.0069 (8)	−0.0046 (9)	−0.0027 (8)
C11	0.0425 (14)	0.0402 (12)	0.0488 (14)	−0.0111 (10)	−0.0074 (11)	0.0119 (10)
C12	0.066 (2)	0.0411 (14)	0.0631 (18)	−0.0116 (13)	−0.0225 (15)	0.0181 (12)
C13	0.0604 (18)	0.0378 (13)	0.0652 (18)	0.0056 (12)	−0.0291 (15)	−0.0014 (12)
C14	0.0394 (14)	0.0497 (14)	0.0588 (17)	0.0077 (11)	−0.0141 (12)	−0.0182 (13)
C15	0.0352 (12)	0.0386 (12)	0.0403 (12)	−0.0010 (9)	−0.0027 (10)	−0.0066 (9)
C16	0.0383 (11)	0.0296 (9)	0.0233 (9)	−0.0041 (8)	0.0085 (8)	0.0026 (7)
C17	0.0517 (15)	0.0365 (12)	0.0567 (16)	0.0037 (11)	0.0191 (13)	0.0110 (11)
C18	0.084 (2)	0.0337 (13)	0.074 (2)	0.0075 (14)	0.0289 (18)	0.0102 (13)
C19	0.112 (3)	0.0328 (13)	0.0425 (15)	−0.0162 (15)	0.0096 (16)	0.0001 (11)
C20	0.075 (2)	0.0490 (15)	0.0475 (15)	−0.0289 (14)	−0.0146 (14)	0.0102 (12)
C21	0.0468 (14)	0.0403 (12)	0.0420 (13)	−0.0126 (11)	−0.0070 (11)	0.0099 (10)
C22	0.0289 (10)	0.0414 (11)	0.0178 (8)	0.0066 (8)	0.0020 (7)	0.0012 (8)
C23	0.0492 (14)	0.0545 (14)	0.0251 (11)	0.0080 (11)	0.0103 (10)	0.0074 (10)
C24	0.0681 (19)	0.081 (2)	0.0200 (11)	0.0268 (16)	0.0089 (12)	0.0097 (12)
C25	0.0566 (17)	0.094 (2)	0.0220 (11)	0.0281 (16)	−0.0077 (11)	−0.0140 (13)
C26	0.0528 (16)	0.084 (2)	0.0350 (13)	0.0006 (15)	−0.0119 (12)	−0.0214 (13)
C27	0.0414 (13)	0.0575 (15)	0.0274 (11)	−0.0076 (11)	0.0006 (10)	−0.0079 (10)
C28	0.0229 (9)	0.0291 (9)	0.0224 (9)	−0.0018 (7)	0.0041 (7)	0.0029 (7)
C29	0.0355 (11)	0.0400 (11)	0.0259 (10)	0.0044 (9)	0.0010 (8)	−0.0040 (8)
C30	0.0416 (13)	0.0431 (13)	0.0412 (13)	0.0099 (10)	0.0048 (10)	−0.0059 (10)
C31	0.0285 (11)	0.0431 (12)	0.0438 (13)	0.0057 (9)	0.0008 (9)	0.0040 (10)
C32	0.0268 (10)	0.0435 (12)	0.0347 (11)	−0.0036 (9)	−0.0055 (9)	0.0044 (9)
C33	0.0252 (10)	0.0372 (10)	0.0265 (10)	−0.0038 (8)	0.0000 (8)	0.0001 (8)
C34	0.0266 (9)	0.0276 (9)	0.0246 (9)	−0.0018 (7)	−0.0010 (8)	−0.0010 (7)
C35	0.0361 (11)	0.0361 (11)	0.0275 (10)	−0.0052 (9)	0.0043 (9)	0.0013 (8)
C36	0.0522 (15)	0.0349 (11)	0.0426 (13)	−0.0109 (10)	0.0076 (11)	0.0048 (10)
C37	0.0611 (16)	0.0270 (11)	0.0519 (15)	−0.0024 (10)	0.0022 (13)	−0.0003 (10)
C38	0.0437 (13)	0.0351 (11)	0.0454 (13)	0.0071 (10)	0.0052 (11)	−0.0061 (10)
C39	0.0301 (10)	0.0344 (11)	0.0376 (11)	0.0006 (8)	0.0032 (9)	−0.0017 (9)
C40	0.0200 (9)	0.0328 (10)	0.0249 (9)	−0.0017 (7)	0.0019 (7)	−0.0026 (7)
C41	0.0283 (10)	0.0364 (11)	0.0312 (10)	−0.0001 (8)	−0.0013 (8)	0.0020 (8)
C42	0.0300 (11)	0.0487 (13)	0.0418 (13)	0.0047 (10)	−0.0083 (10)	−0.0007 (10)
C43	0.0217 (10)	0.0592 (16)	0.0525 (15)	−0.0039 (10)	−0.0046 (10)	−0.0021 (12)
C44	0.0272 (11)	0.0566 (15)	0.0490 (14)	−0.0121 (10)	0.0045 (10)	0.0054 (12)
C45	0.0269 (10)	0.0432 (12)	0.0335 (11)	−0.0055 (9)	0.0023 (9)	0.0059 (9)
C46	0.0219 (9)	0.0353 (10)	0.0196 (8)	−0.0050 (7)	0.0002 (7)	0.0019 (7)
C47	0.0426 (13)	0.0409 (11)	0.0229 (10)	−0.0033 (9)	0.0051 (9)	−0.0012 (8)
C48	0.0562 (16)	0.0634 (16)	0.0194 (10)	−0.0107 (13)	0.0062 (10)	−0.0019 (10)
C49	0.0447 (14)	0.0647 (16)	0.0231 (10)	−0.0129 (12)	−0.0038 (10)	0.0123 (10)
C50	0.0359 (12)	0.0485 (13)	0.0337 (11)	−0.0026 (10)	−0.0046 (9)	0.0121 (10)
C51	0.0313 (10)	0.0383 (11)	0.0252 (10)	0.0002 (8)	0.0009 (8)	0.0037 (8)
O1	0.134 (7)	0.166 (9)	0.181 (10)	0.025 (6)	−0.056 (6)	−0.129 (9)
O1'	0.112 (6)	0.267 (13)	0.066 (4)	−0.075 (7)	−0.053 (4)	0.045 (5)

### Geometric parameters (Å, °)

**Table:**

Ru1—C1	1.8571 (19)	C30—H30A	0.9500
Ru1—Cl2	2.3498 (5)	C31—C32	1.387 (3)
Ru1—Cl1	2.3639 (5)	C31—H31A	0.9500
Ru1—P1	2.3863 (5)	C32—C33	1.389 (3)
Ru1—P2	2.4087 (5)	C32—H32A	0.9500
P1—C22	1.822 (2)	C33—H33A	0.9500
P1—C16	1.824 (2)	C34—C35	1.394 (3)
P1—C28	1.834 (2)	C34—C39	1.401 (3)
P2—C40	1.827 (2)	C35—C36	1.392 (3)
P2—C34	1.833 (2)	C35—H35A	0.9500
P2—C46	1.8371 (19)	C36—C37	1.384 (4)
C1—C2	1.465 (3)	C36—H36A	0.9500
C1—C9	1.494 (3)	C37—C38	1.380 (4)
C2—C3	1.362 (3)	C37—H37A	0.9500
C2—H2A	0.9500	C38—C39	1.389 (3)
C3—C10	1.468 (3)	C38—H38A	0.9500
C3—C4	1.484 (3)	C39—H39A	0.9500
C4—C5	1.382 (3)	C40—C45	1.391 (3)
C4—C9	1.416 (3)	C40—C41	1.396 (3)
C5—C6	1.403 (4)	C41—C42	1.386 (3)
C5—H5A	0.9500	C41—H41A	0.9500
C6—C7	1.383 (4)	C42—C43	1.383 (4)
C6—H6A	0.9500	C42—H42A	0.9500
C7—C8	1.403 (3)	C43—C44	1.375 (4)
C7—H7A	0.9500	C43—H43A	0.9500
C8—C9	1.385 (3)	C44—C45	1.392 (3)
C8—H8A	0.9500	C44—H44A	0.9500
C10—C15	1.394 (3)	C45—H45A	0.9500
C10—C11	1.394 (3)	C46—C47	1.391 (3)
C11—C12	1.392 (4)	C46—C51	1.392 (3)
C11—H11A	0.9500	C47—C48	1.394 (3)
C12—C13	1.379 (5)	C47—H47A	0.9500
C12—H12A	0.9500	C48—C49	1.377 (4)
C13—C14	1.375 (4)	C48—H48A	0.9500
C13—H13A	0.9500	C49—C50	1.380 (4)
C14—C15	1.390 (4)	C49—H49A	0.9500
C14—H14A	0.9500	C50—C51	1.390 (3)
C15—H15A	0.9500	C50—H50A	0.9500
C16—C21	1.385 (3)	C51—H51A	0.9500
C16—C17	1.398 (3)	O1—C55	1.344 (8)
C17—C18	1.388 (4)	O1—C52	1.402 (8)
C17—H17A	0.9500	C52—C53	1.535 (14)
C18—C19	1.388 (5)	C52—H52A	0.9900
C18—H18A	0.9500	C52—H52B	0.9900
C19—C20	1.374 (5)	C53—C54	1.467 (9)
C19—H19A	0.9500	C53—H53A	0.9900
C20—C21	1.397 (3)	C53—H53B	0.9900
C20—H20A	0.9500	C54—C55	1.54 (2)
C21—H21A	0.9500	C54—H54A	0.9900
C22—C27	1.386 (3)	C54—H54B	0.9900
C22—C23	1.393 (3)	C55—H55A	0.9900
C23—C24	1.397 (4)	C55—H55B	0.9900
C23—H23A	0.9500	O1'—C52'	1.362 (8)
C24—C25	1.366 (5)	O1'—C55'	1.414 (8)
C24—H24A	0.9500	C52'—C53'	1.543 (9)
C25—C26	1.373 (5)	C52'—H52C	0.9900
C25—H25A	0.9500	C52'—H52D	0.9900
C26—C27	1.395 (3)	C53'—C54'	1.436 (13)
C26—H26A	0.9500	C53'—H53C	0.9900
C27—H27A	0.9500	C53'—H53D	0.9900
C28—C29	1.396 (3)	C54'—C55'	1.489 (14)
C28—C33	1.400 (3)	C54'—H54C	0.9900
C29—C30	1.391 (3)	C54'—H54D	0.9900
C29—H29A	0.9500	C55'—H55C	0.9900
C30—C31	1.392 (3)	C55'—H55D	0.9900
			
C1—Ru1—Cl2	102.65 (6)	C32—C31—H31A	120.2
C1—Ru1—Cl1	99.62 (6)	C30—C31—H31A	120.2
Cl2—Ru1—Cl1	157.71 (2)	C31—C32—C33	120.6 (2)
C1—Ru1—P1	93.64 (6)	C31—C32—H32A	119.7
Cl2—Ru1—P1	89.908 (18)	C33—C32—H32A	119.7
Cl1—Ru1—P1	89.691 (17)	C32—C33—C28	120.5 (2)
C1—Ru1—P2	99.33 (6)	C32—C33—H33A	119.7
Cl2—Ru1—P2	89.461 (18)	C28—C33—H33A	119.7
Cl1—Ru1—P2	85.934 (17)	C35—C34—C39	118.75 (19)
P1—Ru1—P2	166.829 (18)	C35—C34—P2	119.57 (16)
C22—P1—C16	104.79 (10)	C39—C34—P2	121.62 (16)
C22—P1—C28	102.25 (9)	C36—C35—C34	120.4 (2)
C16—P1—C28	106.53 (10)	C36—C35—H35A	119.8
C22—P1—Ru1	115.80 (7)	C34—C35—H35A	119.8
C16—P1—Ru1	105.53 (7)	C37—C36—C35	120.2 (2)
C28—P1—Ru1	120.65 (6)	C37—C36—H36A	119.9
C40—P2—C34	100.49 (9)	C35—C36—H36A	119.9
C40—P2—C46	104.10 (9)	C38—C37—C36	120.0 (2)
C34—P2—C46	106.40 (9)	C38—C37—H37A	120.0
C40—P2—Ru1	121.25 (7)	C36—C37—H37A	120.0
C34—P2—Ru1	106.92 (7)	C37—C38—C39	120.3 (2)
C46—P2—Ru1	115.83 (7)	C37—C38—H38A	119.8
C2—C1—C9	104.83 (16)	C39—C38—H38A	119.8
C2—C1—Ru1	124.67 (14)	C38—C39—C34	120.3 (2)
C9—C1—Ru1	130.29 (15)	C38—C39—H39A	119.9
C3—C2—C1	110.91 (17)	C34—C39—H39A	119.9
C3—C2—H2A	124.5	C45—C40—C41	118.98 (19)
C1—C2—H2A	124.5	C45—C40—P2	122.07 (16)
C2—C3—C10	125.34 (18)	C41—C40—P2	118.92 (15)
C2—C3—C4	108.29 (18)	C42—C41—C40	120.3 (2)
C10—C3—C4	126.32 (17)	C42—C41—H41A	119.8
C5—C4—C9	120.9 (2)	C40—C41—H41A	119.8
C5—C4—C3	131.2 (2)	C43—C42—C41	120.3 (2)
C9—C4—C3	107.73 (16)	C43—C42—H42A	119.9
C4—C5—C6	118.4 (2)	C41—C42—H42A	119.9
C4—C5—H5A	120.8	C44—C43—C42	119.8 (2)
C6—C5—H5A	120.8	C44—C43—H43A	120.1
C7—C6—C5	120.8 (2)	C42—C43—H43A	120.1
C7—C6—H6A	119.6	C43—C44—C45	120.5 (2)
C5—C6—H6A	119.6	C43—C44—H44A	119.7
C6—C7—C8	121.1 (2)	C45—C44—H44A	119.7
C6—C7—H7A	119.4	C40—C45—C44	120.1 (2)
C8—C7—H7A	119.4	C40—C45—H45A	119.9
C9—C8—C7	118.3 (2)	C44—C45—H45A	119.9
C9—C8—H8A	120.8	C47—C46—C51	119.14 (19)
C7—C8—H8A	120.8	C47—C46—P2	122.31 (16)
C8—C9—C4	120.43 (18)	C51—C46—P2	118.54 (15)
C8—C9—C1	131.43 (19)	C46—C47—C48	119.9 (2)
C4—C9—C1	107.96 (17)	C46—C47—H47A	120.1
C15—C10—C11	118.4 (2)	C48—C47—H47A	120.1
C15—C10—C3	120.6 (2)	C49—C48—C47	120.6 (2)
C11—C10—C3	121.0 (2)	C49—C48—H48A	119.7
C12—C11—C10	120.7 (3)	C47—C48—H48A	119.7
C12—C11—H11A	119.7	C48—C49—C50	119.8 (2)
C10—C11—H11A	119.7	C48—C49—H49A	120.1
C13—C12—C11	120.1 (3)	C50—C49—H49A	120.1
C13—C12—H12A	120.0	C49—C50—C51	120.1 (2)
C11—C12—H12A	120.0	C49—C50—H50A	119.9
C14—C13—C12	119.9 (3)	C51—C50—H50A	119.9
C14—C13—H13A	120.0	C50—C51—C46	120.4 (2)
C12—C13—H13A	120.0	C50—C51—H51A	119.8
C13—C14—C15	120.5 (3)	C46—C51—H51A	119.8
C13—C14—H14A	119.8	C55—O1—C52	105.0 (8)
C15—C14—H14A	119.8	O1—C52—C53	111.0 (7)
C14—C15—C10	120.5 (2)	O1—C52—H52A	109.4
C14—C15—H15A	119.8	C53—C52—H52A	109.4
C10—C15—H15A	119.8	O1—C52—H52B	109.4
C21—C16—C17	118.8 (2)	C53—C52—H52B	109.4
C21—C16—P1	124.34 (18)	H52A—C52—H52B	108.0
C17—C16—P1	116.82 (18)	C54—C53—C52	106.8 (11)
C18—C17—C16	120.5 (3)	C54—C53—H53A	110.4
C18—C17—H17A	119.8	C52—C53—H53A	110.4
C16—C17—H17A	119.8	C54—C53—H53B	110.4
C17—C18—C19	120.1 (3)	C52—C53—H53B	110.4
C17—C18—H18A	120.0	H53A—C53—H53B	108.6
C19—C18—H18A	120.0	C53—C54—C55	99.9 (11)
C20—C19—C18	119.9 (3)	C53—C54—H54A	111.8
C20—C19—H19A	120.0	C55—C54—H54A	111.8
C18—C19—H19A	120.0	C53—C54—H54B	111.8
C19—C20—C21	120.2 (3)	C55—C54—H54B	111.8
C19—C20—H20A	119.9	H54A—C54—H54B	109.5
C21—C20—H20A	119.9	O1—C55—C54	117.1 (10)
C16—C21—C20	120.6 (3)	O1—C55—H55A	108.0
C16—C21—H21A	119.7	C54—C55—H55A	108.0
C20—C21—H21A	119.7	O1—C55—H55B	108.0
C27—C22—C23	118.9 (2)	C54—C55—H55B	108.0
C27—C22—P1	119.02 (16)	H55A—C55—H55B	107.3
C23—C22—P1	122.00 (18)	C52'—O1'—C55'	111.5 (8)
C22—C23—C24	119.9 (3)	O1'—C52'—C53'	107.4 (9)
C22—C23—H23A	120.0	O1'—C52'—H52C	110.2
C24—C23—H23A	120.0	C53'—C52'—H52C	110.2
C25—C24—C23	120.6 (3)	O1'—C52'—H52D	110.2
C25—C24—H24A	119.7	C53'—C52'—H52D	110.2
C23—C24—H24A	119.7	H52C—C52'—H52D	108.5
C24—C25—C26	119.9 (2)	C54'—C53'—C52'	102.8 (8)
C24—C25—H25A	120.1	C54'—C53'—H53C	111.2
C26—C25—H25A	120.1	C52'—C53'—H53C	111.2
C25—C26—C27	120.4 (3)	C54'—C53'—H53D	111.2
C25—C26—H26A	119.8	C52'—C53'—H53D	111.2
C27—C26—H26A	119.8	H53C—C53'—H53D	109.1
C22—C27—C26	120.2 (2)	C53'—C54'—C55'	110.0 (7)
C22—C27—H27A	119.9	C53'—C54'—H54C	109.7
C26—C27—H27A	119.9	C55'—C54'—H54C	109.7
C29—C28—C33	118.46 (19)	C53'—C54'—H54D	109.7
C29—C28—P1	120.43 (15)	C55'—C54'—H54D	109.7
C33—C28—P1	120.87 (15)	H54C—C54'—H54D	108.2
C30—C29—C28	120.9 (2)	O1'—C55'—C54'	104.7 (8)
C30—C29—H29A	119.5	O1'—C55'—H55C	110.8
C28—C29—H29A	119.5	C54'—C55'—H55C	110.8
C29—C30—C31	120.0 (2)	O1'—C55'—H55D	110.8
C29—C30—H30A	120.0	C54'—C55'—H55D	110.8
C31—C30—H30A	120.0	H55C—C55'—H55D	108.9
C32—C31—C30	119.5 (2)		
			
C1—Ru1—P1—C22	−74.82 (10)	C16—P1—C22—C27	158.88 (19)
Cl2—Ru1—P1—C22	27.84 (8)	C28—P1—C22—C27	−90.12 (19)
Cl1—Ru1—P1—C22	−174.44 (8)	Ru1—P1—C22—C27	43.1 (2)
P2—Ru1—P1—C22	115.08 (10)	C16—P1—C22—C23	−24.7 (2)
C1—Ru1—P1—C16	169.79 (9)	C28—P1—C22—C23	86.3 (2)
Cl2—Ru1—P1—C16	−87.55 (8)	Ru1—P1—C22—C23	−140.47 (17)
Cl1—Ru1—P1—C16	70.17 (8)	C27—C22—C23—C24	0.6 (4)
P2—Ru1—P1—C16	−0.30 (11)	P1—C22—C23—C24	−175.90 (19)
C1—Ru1—P1—C28	49.26 (9)	C22—C23—C24—C25	1.2 (4)
Cl2—Ru1—P1—C28	151.93 (8)	C23—C24—C25—C26	−1.5 (4)
Cl1—Ru1—P1—C28	−50.36 (8)	C24—C25—C26—C27	0.0 (4)
P2—Ru1—P1—C28	−120.83 (10)	C23—C22—C27—C26	−2.0 (4)
C1—Ru1—P2—C40	70.80 (10)	P1—C22—C27—C26	174.5 (2)
Cl2—Ru1—P2—C40	−31.93 (8)	C25—C26—C27—C22	1.8 (4)
Cl1—Ru1—P2—C40	169.90 (8)	C22—P1—C28—C29	11.09 (19)
P1—Ru1—P2—C40	−119.22 (10)	C16—P1—C28—C29	120.79 (17)
C1—Ru1—P2—C34	−175.18 (9)	Ru1—P1—C28—C29	−119.18 (16)
Cl2—Ru1—P2—C34	82.09 (7)	C22—P1—C28—C33	−174.59 (16)
Cl1—Ru1—P2—C34	−76.08 (7)	C16—P1—C28—C33	−64.90 (18)
P1—Ru1—P2—C34	−5.20 (10)	Ru1—P1—C28—C33	55.14 (18)
C1—Ru1—P2—C46	−56.81 (9)	C33—C28—C29—C30	−1.6 (3)
Cl2—Ru1—P2—C46	−159.54 (7)	P1—C28—C29—C30	172.83 (18)
Cl1—Ru1—P2—C46	42.29 (7)	C28—C29—C30—C31	−0.2 (4)
P1—Ru1—P2—C46	113.17 (10)	C29—C30—C31—C32	1.6 (4)
Cl2—Ru1—C1—C2	−144.70 (15)	C30—C31—C32—C33	−1.2 (3)
Cl1—Ru1—C1—C2	36.33 (16)	C31—C32—C33—C28	−0.7 (3)
P1—Ru1—C1—C2	−53.98 (15)	C29—C28—C33—C32	2.1 (3)
P2—Ru1—C1—C2	123.74 (15)	P1—C28—C33—C32	−172.32 (16)
Cl2—Ru1—C1—C9	41.46 (17)	C40—P2—C34—C35	137.09 (17)
Cl1—Ru1—C1—C9	−137.51 (16)	C46—P2—C34—C35	−114.69 (17)
P1—Ru1—C1—C9	132.19 (16)	Ru1—P2—C34—C35	9.67 (18)
P2—Ru1—C1—C9	−50.09 (17)	C40—P2—C34—C39	−40.11 (19)
C9—C1—C2—C3	5.2 (2)	C46—P2—C34—C39	68.11 (19)
Ru1—C1—C2—C3	−169.96 (14)	Ru1—P2—C34—C39	−167.54 (16)
C1—C2—C3—C10	172.22 (19)	C39—C34—C35—C36	−1.1 (3)
C1—C2—C3—C4	−5.4 (2)	P2—C34—C35—C36	−178.34 (18)
C2—C3—C4—C5	−171.0 (2)	C34—C35—C36—C37	−1.3 (4)
C10—C3—C4—C5	11.3 (4)	C35—C36—C37—C38	1.7 (4)
C2—C3—C4—C9	3.5 (2)	C36—C37—C38—C39	0.3 (4)
C10—C3—C4—C9	−174.12 (19)	C37—C38—C39—C34	−2.7 (4)
C9—C4—C5—C6	1.4 (3)	C35—C34—C39—C38	3.0 (3)
C3—C4—C5—C6	175.3 (2)	P2—C34—C39—C38	−179.75 (18)
C4—C5—C6—C7	−0.4 (4)	C34—P2—C40—C45	131.75 (18)
C5—C6—C7—C8	−1.2 (4)	C46—P2—C40—C45	21.7 (2)
C6—C7—C8—C9	1.8 (3)	Ru1—P2—C40—C45	−110.96 (17)
C7—C8—C9—C4	−0.8 (3)	C34—P2—C40—C41	−50.32 (18)
C7—C8—C9—C1	−175.4 (2)	C46—P2—C40—C41	−160.35 (17)
C5—C4—C9—C8	−0.7 (3)	Ru1—P2—C40—C41	66.97 (18)
C3—C4—C9—C8	−175.93 (18)	C45—C40—C41—C42	−0.1 (3)
C5—C4—C9—C1	174.97 (19)	P2—C40—C41—C42	−178.08 (18)
C3—C4—C9—C1	−0.2 (2)	C40—C41—C42—C43	0.5 (4)
C2—C1—C9—C8	172.2 (2)	C41—C42—C43—C44	−0.8 (4)
Ru1—C1—C9—C8	−13.0 (3)	C42—C43—C44—C45	0.8 (4)
C2—C1—C9—C4	−2.8 (2)	C41—C40—C45—C44	0.0 (3)
Ru1—C1—C9—C4	171.94 (14)	P2—C40—C45—C44	177.98 (19)
C2—C3—C10—C15	45.9 (3)	C43—C44—C45—C40	−0.4 (4)
C4—C3—C10—C15	−136.9 (2)	C40—P2—C46—C47	74.32 (19)
C2—C3—C10—C11	−130.8 (2)	C34—P2—C46—C47	−31.3 (2)
C4—C3—C10—C11	46.5 (3)	Ru1—P2—C46—C47	−149.97 (16)
C15—C10—C11—C12	−0.5 (4)	C40—P2—C46—C51	−105.03 (17)
C3—C10—C11—C12	176.3 (2)	C34—P2—C46—C51	149.34 (16)
C10—C11—C12—C13	1.2 (4)	Ru1—P2—C46—C51	30.68 (18)
C11—C12—C13—C14	−1.2 (4)	C51—C46—C47—C48	0.3 (3)
C12—C13—C14—C15	0.4 (4)	P2—C46—C47—C48	−179.06 (18)
C13—C14—C15—C10	0.4 (4)	C46—C47—C48—C49	0.2 (4)
C11—C10—C15—C14	−0.4 (3)	C47—C48—C49—C50	−0.2 (4)
C3—C10—C15—C14	−177.1 (2)	C48—C49—C50—C51	−0.3 (4)
C22—P1—C16—C21	112.0 (2)	C49—C50—C51—C46	0.7 (3)
C28—P1—C16—C21	4.1 (2)	C47—C46—C51—C50	−0.7 (3)
Ru1—P1—C16—C21	−125.32 (19)	P2—C46—C51—C50	178.67 (17)
C22—P1—C16—C17	−71.0 (2)	C55—O1—C52—C53	−1.5 (14)
C28—P1—C16—C17	−178.87 (18)	O1—C52—C53—C54	4.5 (16)
Ru1—P1—C16—C17	51.76 (19)	C52—C53—C54—C55	−4.9 (16)
C21—C16—C17—C18	0.9 (4)	C52—O1—C55—C54	−2.0 (17)
P1—C16—C17—C18	−176.3 (2)	C53—C54—C55—O1	5(2)
C16—C17—C18—C19	−0.1 (4)	C55'—O1'—C52'—C53'	−20.0 (16)
C17—C18—C19—C20	−1.0 (5)	O1'—C52'—C53'—C54'	15.0 (15)
C18—C19—C20—C21	1.3 (4)	C52'—C53'—C54'—C55'	−5.1 (14)
C17—C16—C21—C20	−0.6 (4)	C52'—O1'—C55'—C54'	16.4 (14)
P1—C16—C21—C20	176.4 (2)	C53'—C54'—C55'—O1'	−5.9 (13)
C19—C20—C21—C16	−0.5 (4)		

### Hydrogen-bond geometry (Å, °)

**Table:**

*D*—H···*A*	*D*—H	H···*A*	*D*···*A*	*D*—H···*A*
C2—H2A···C28	0.95	2.59	3.271 (3)	128
C2—H2A···C33	0.95	2.63	3.522 (3)	156
C51—H51A···C1	0.95	2.59	3.448 (3)	150
C8—H8A···Cl2	0.95	2.80	3.497 (2)	131
C17—H17A···Cl2	0.95	2.80	3.640 (3)	148
C33—H33A···Cl1	0.95	2.66	3.359 (2)	131
C48—H48A···Cl2^i^	0.95	2.83	3.699 (2)	153
C25—H25A···Cg1^ii^	0.95	2.88	3.727 (3)	149
C30—H30A···Cg2^iii^	0.95	2.98	3.751 (3)	139

Symmetry codes:  (i) *x*, −*y*+1/2, *z*−1/2; (ii) *x*, −*y*+1/2, *z*+1/2; (iii) −*x*, *y*+1/2, −*z*+1/2.
